# The Relationship between Burden and Depression in Spouses of Chronic Kidney Disease Patients

**DOI:** 10.1155/2018/8694168

**Published:** 2018-05-13

**Authors:** Athina Paschou, Dimitrios Damigos, Petros Skapinakis, Kostas Siamopoulos

**Affiliations:** Faculty of Medicine, School of Health Sciences, University of Ioannina, Ioannina, Greece

## Abstract

The purpose of the present study was to investigate the burden and depression in spouses of patients with chronic kidney disease (CKD). The interrelation between burden and depression in family caregivers has been pointed out by previous researches in several chronic diseases and researchers agree that they clearly go together and one cannot talk about one without considering the other. More particularly, in the present study, the caregiver burden, the depression, anxiety, and also health-related quality of life and demographic factors of spouses of patients with CKD were examined, using self-report questionnaires. Participants were 50 spouses of patients with CKD, 29 of whom were dialysis dependent and 21 were not dialysis dependent. Group differences were examined for participants. Results confirm the interrelation between caregiver burden and depression in spouses. The increased perceived burden related to higher levels of depression. Low levels of caregiver burden, depression, anxiety, and satisfactory quality of life were found in spouses, with no differences between them relevant to whether the patients were dialysis dependent or not. All the above parameters interrelated. Implications for the findings and future research directions are discussed.

## 1. Introduction

Chronic disease affects patients and their family thoroughly, since the family and especially the spouse constitute the ultimate care unit [1–3], which is also the case in Greece [4]. Living with a chronic disease patient and being the main caregiver can be very stressful. Feelings of depression, anxiety, and burden are often reported in this population and they are interrelated [3, 5, 6] while worse health status has also been pointed out [7].

The caregiver burden may be more of a universal phenomenon [8] rather than disease-specific or related to patient variables. Regardless of the type of the disease, it is a multidimensional phenomenon affecting caregivers physically, emotionally, and socially [9]. Findings of previous studies highlight the need to focus on the health of caregivers, since it is possible that they are even more influenced emotionally from the chronic disease than the patient [10]. As Adelman et al. point out [11], caregivers become the invisible patient and therefore physicians must recognize the importance of family caregiving since the health of their patients depends on the quality of home-based caregiving.

A factor which has been related to caregiver burden is depression. Over the last two decades, depression has been identified as both a risk factor for and an outcome of caregiver burden [12], a remark confirmed also by more recent studies [11]. As researchers have pointed out [13], burden and depression go hand-in-hand and they are considered to be either virtually synonymous [14] or unique constructs [13–16]. The relationship between them has been verified in studies in different cultural environments [17] and in various chronic diseases, like cancer, Alzheimer, and stroke [9, 15, 18, 19], irrelevant to the patients' or caregivers' characteristics [8, 19].

In order to develop strategies which may benefit burdened spouses caregivers, identification of other characteristics, such as quality of life (QOL) [20] and marital satisfaction, is important. Marriage has been found to relate to good health for the couple members [21] but relationship satisfaction is the key factor for this positive effect. Higher levels of marital quality have been related to more adaptive illness perceptions and illness management behavior, as well as to better health and QOL for both patients and their partners [22–26].

In particular, in chronic kidney disease (CKD) researchers point out that the CKD patients function within a psychosocial dyad [27] with their spouse and in some cases within a depressive dyad [28]. The effect of marital relationship on the health status of the couple members has been thoroughly studied in the case of chronic kidney disease (CKD) [29, 30]. The majority of studies concerning the caregiver burden of family caregivers of patients with CKD focus on patients at the end stage renal disease and patients in hemodialysis [31–33], but they do not necessarily focus on spouses as caregivers. It has been pointed out that caregivers of hemodialysis patients experience burden, which affects their quality of life negatively and that the emotional aspects especially of female spouses caregivers and patients predict burden [34]. Although previous studies examined spousal relationship in end stage renal disease or transplantation patients, interventions in earlier stages of the disease remain unexplored [35–39].

The purpose of this study was to investigate the relationship between caregiver burden and depression in spouses of CKD patients and to clarify the role of anxiety, quality of life, and demographic factors in this relationship. Specifically, whether the caregiver burden depends on the above parameters was investigated. The following research questions were examined:

1. Which are the levels of burden, depression, anxiety, and QOL in spouses of patients with CKD?
2. Is the relationship between burden and depression verified in the present study group, regardless of demographic factors and spouses' or patients' characteristics?

## 2. Materials and Methods

### 2.1. Study Design and Participants

The present study was conducted between September 2013 and June 2015 at the Nephrology Department of the University Hospital of Ioannina, Greece. Participants were spouses of patients with CKD who received medical care in the units of the Nephrology Department of the University Hospital of Ioannina (Outpatients Clinics, Renal Ward, Hemodialysis Unit, and Peritoneal Dialysis Unit). The caregiver burden, depression, anxiety, health-related quality of life, and important demographic factors like common health problems were evaluated in spouses. All variables were measured with self-report questionnaires, which are often used in clinical practice for estimating the psychological burden [40].

The spouses of patients with CKD were recruited via three different ways: (1) via approaching in person, while accompanying their spouse at the hospital, (2) via the CKD patients during their regular appointments, and (3) via phone. Spouses were purposively sampled to include patients from all units of the Nephrology Department. Participants were eligible if they are (1) spouse of the patient, (2) aged >18 years, (3) able to understand and communicate in Greek, and (4) without severe current health problems which would prevent them from understanding and answering the questionnaires. If the spouses asked for assistance to complete the questionnaires, the data collector (first author) conducted interviews in vivo (at the hospital) or by phone (at the Laboratory of Medical Psychology, Faculty of Medicine, University of Ioannina). Interviews were carried out in a private room and lasted between 50–60 minutes. Finally, 33 spouses completed the questionnaires alone and 17 with the help of the data collector.

All spouses of CKD patients were approached to participate in the present study. Eligible participants, who agreed to take part and signed a written consent, were 50 spouses (12 males and 38 females). They were divided into two groups, namely, spouses of dialysis dependent patients (*n* = 29) and spouses of patients not dialysis dependent (*n* = 21). An ethical approval for the study was obtained from the scientific committee of the University Hospital of Ioannina.

### 2.2. Measures

#### 2.2.1. Assessment of Quality of Life and Other Sociodemographic Factors

Health-related quality of life was measured with the EuroQol EQ-5D. EQ-5D is a standardized health-related quality of life questionnaire developed by the EuroQol Group [41] and it has been validated in Greece by Kontodimopoulos et al. [42]. It consists of 2 parts: the EQ-5D descriptive system and the EQ VAS. The EQ VAS records the respondent's self-rated health on a visual analogue scale where the endpoints are labelled “best imaginable health state” and “worst imaginable health state.” This information can be used as a quantitative measure of health outcome as judged by the individual respondents. A scoring algorithm for the EQ-5D index descriptive system is used in studies in Greece [43] as well as in the present study.

Additionally, spouses checked on a list of common health problems and if they suffered from any, they answered in a VAS about their marital satisfaction on a range from 1 to 10 and they completed a questionnaire with important demographic factors.

#### 2.2.2. Caregiver Burden

Caregiver burden was measured with the Zarit Burden Interview (ZBI). This scale is designed to evaluate the stress of the caregivers. The respondents answer 22 questions relevant to the effect of the patient's incapability of their life. Item responses range between never (0), rarely (1), sometimes (2), relatively often (3), and almost always (4). Zarit Index scores range from 0 to 88 and are calculated by summing across the 22 items. The scores can be categorized into level of burden severity: not at all to moderate (0–20), mild to moderate (21–40), moderate to severe (41–60), and severe (61–88) [44]. The Greek translation and validation were introduced by Papastavrou et al. [45] and the internal consistency reliability was high (*a* = 0.93). To adjust it to the participants of the present study, the items were rephrased to refer to “spouse” of the patient.

#### 2.2.3. Assessment of Depression

Depression was measured with a reliable and valid questionnaire for the assessment of depression, which derived from the Diagnostic and Statistical Manual of Mental Disorders, 4th Edition [32] and the Patient Health Questionnaire-9 (PHQ-9). The PHQ-9 consists of 9 items, with each item referring to symptoms of depression over the previous weeks. Item responses range between not at all (0), on several days (1), on more than half of the days (2), and nearly every day (3). PHQ-9 Severity Index scores range from 0 to 27 and are calculated by summing across the 9 items. PHQ-9 Severity Index scores can be categorized into level of depression severity: none (0–4), mild (5–9), moderate (10–14), moderately severe (15–19), and severe (20–27) [46]. Alternatively, a cut-off point at score 10 can also be used to distinguish the existence and the absence of depression [47].

#### 2.2.4. Assessment of Anxiety

Anxiety was measured with Generalized Anxiety Disorder (GAD- 2) questionnaire. The 2-item form derived from GAD-7 and it was as informative as the 7-item form for GAD and other anxiety disorders. Kroenke et al. [48] attempted to create an anxiety disturbance detection questionnaire with two questions (GAD-2). These questions were selected because they relate to nuclear and more general symptoms compared with those of the questionnaire GAD-7. The answers are scored from 0 (not at all) to 3 (nearly every day). It was found that GAD-2 with a score ≥ 3 detects stress disorders with acceptable levels sensitivity (70–86%) and specificity (81–83%) [40]. According to Skapinakis [49] the authors' conclusion that the 2-item scale could be used as a brief screening tool is well justified by the data, especially for generalized anxiety and possibly for panic disorder.

### 2.3. Statistical Analysis

We used the survey commands in Stata version 10.0 to calculate prevalence estimates and 95% confidence intervals [50]. These commands take into account the complex sampling design. Associations between spouse burden and depression, stage of chronic kidney disease, age, sex, marital satisfaction, education, and employment were calculated at 95% confidence intervals with multiple linear regression models using the survey commands in Stata 10.0. All evaluations of statistical significance are based on two-sided tests using the 5% level of significance.

## 3. Results

### 3.1. Description of the Sample

For the present research, 82 spouses of chronic kidney disease patients were approached to participate. From them, 32 denied to participate or did not return the questionnaires. Finally, 50 spouses completed the questionnaires. The spouses were divided into two groups, spouses of dialysis dependent patients (*n* = 29, mean age 60.69 years, and SD 11.13) and spouses of patients not dialysis dependent (*n* = 21, mean age 63.43 years, and SD 11.56).

The patients have been suffering from CKD for 8.76 years (SD 7.26) and their age was 65.48 years, SD 10.54 (dialysis dependent patients), and 66.09 years, SD 9.62 (patients not dialysis dependent). Concerning the severity of CKD, 13 patients suffered from CKD stages I–IV (their spouses were approached at the Outpatients Clinics), 8 patients were at stage V of CKD (their spouses were approached at the Renal Ward), and 29 patients suffered from CKD at the final stage (their spouses were approached at the Hemodialysis Unit and Peritoneal Dialysis Unit), 9 of whom were on hemodialysis and 20 underwent peritoneal dialysis.

Spouses have been a couple with their partner for 38.3 years, SD 11.7. Most spouses had monthly income between 501€ and 1000€ (*n* = 22, 44.0%) and the next bigger category was between 1001€ and 2000€ (*n* = 16, 32.0%). Most spouses reported none or a few financial difficulties (*n* = 29, 58.0%). Based on the VAS used to describe their marital satisfaction, spouses reported being highly satisfied (8.46, SD 1.51). The only parameter where the two groups differed was the educational level (Pr = 0.052). The spouses of dialysis dependent patients had received mainly primary education (*n* = 19) whereas spouses of patients not dialysis dependent were divided almost equally in the three different educational groups (primary education, high school, and higher education).

The most common self-reported health problems in the group of spouses were high levels of cholesterol (*n* = 23), hypertension (*n* = 16), diabetes mellitus (*n* = 12), musculoskeletal pain (*n* = 14, of which 12 were spouses of patients not dialysis dependent), and thyroid disease (*n* = 8). Only 8 participants reported no health problems, 5 of which were spouses of dialysis dependent patients.

Table 1 includes complete demographic characteristics of spouses divided into the two groups, by sex and basic information about the patients.

### 3.2. Levels of Caregiver Burden, Depression, and Anxiety in Spouses

Results of the present study do not indicate high levels of burden and psychological distress in spouses, regardless of whether the patient was dialysis dependent or not. Specifically, the mean (SD) score of caregiver burden in total sample was 27.26 (18.33), which indicates moderate burden in spouses. The mean (SD) score of depression in total sample was 5.64 (4.80), which indicates no to marginally mild depression and finally the mean (SD) anxiety score in total sample was 1.90 (1.64) which does not indicate any stress disorder. Despite the fact that spouses of dialysis dependent patients and spouses of not dialysis dependent patients did not differ statistically in the severity of the above parameters, spouses of dialysis dependent patients scored higher in all variables.  Table 2 presents all the scores in total sample and in two groups.

Table 3 presents the correlation coefficients between caregiver burden, depression, anxiety, and also health-related quality of life in spouses. As expected, the above parameters strongly correlated with each other. Depression and caregiver burden correlated significantly (*p* ≤ 0.001). Higher levels of both depression and anxiety relate to higher levels of caregiver burden.

### 3.3. Evaluation of Health-Related Quality of Life in Spouses

The health-related quality of life in spouses in the present study, as indicated by EQ5D-index, was similar to and not worse than that in the general Greek population [37, 38]. The mean (SD) value in total sample was 0.747 (2.21). The two groups were found to differ significantly (*p* = 0.042) only in the self-evaluation of state of health, as measured with EQ5D-VAS, where spouses of not dialysis dependent patients evaluated their total health as better, mean (SD) 76.57 (16.15), than spouses of dialysis dependent patients, 66.72 (16.60). The above findings are presented in Table 3. As indicated by correlations coefficients, health-related quality of life worsens as levels of burden, depression, and anxiety increase (Table 3). Concerning the five dimensions of EQ5D (mobility, self-care, usual activities, pain/discomfort, and anxiety/depression), the spouses reported having no problems (80%–100%) in the first three dimensions. In the dimension “pain/discomfort,” 27 spouses (54%) reported moderate pain/discomfort and in the last dimension 29 spouses (58%) reported being moderately anxious/depressed.

Multiple linear regression analysis was conducted to investigate independent associations of many variables with spouse caregiver burden. The independent variables (stage of CKD, age, sex, marital satisfaction, education, employment, and depression) were regressed upon spouse caregiver burden (Table 4). Depression (*B* = 2.57, *p* ≤ 0.001) remained in the final regression model as predictor of spouse caregiver burden. Higher spouse caregiver burden scores were predicted by depression, measured by PHQ 9.

## 4. Discussion

In this study of 50 spouses caregivers of chronic kidney disease patients, the relationship between caregiver burden, depression, quality of life, anxiety, and marital satisfaction of the spouses was investigated. We confirmed earlier studies with our findings of association of caregiver burden and depression. Higher spouse caregiver burden scores were predicted by depression. Caregiver burden, depression, anxiety, and health-related QOL interrelated but their mean scores were relatively low in spouses. The severity of chronic kidney disease, dialysis dependent versus not dialysis dependent, did not affect the results. This finding agrees with previous studies [8, 9, 16–18].

In previous studies, patients and caregivers had similar psychological health status [51] and even though the total perceived burden of both groups was relatively low, an important percentage of participants met the criteria for depression. The low levels of depression in spouses in the present study are in accordance with the low levels of depression in CKD patients of the same Nephrology Department (University Hospital of Ioannina), found in the study of Ikonomou et al. [52]. This agrees with the remark of Khaira et al. [28] that CKD patients who are dialysis dependent and their spouses interact in terms of depression levels.

A possible explanation of the good image presented from the spouses of patients with CKD in the present study could be relevant to the high duration of their spouse's disease. Karademas [2] indicates the constant dynamic interaction between patients and their spouses in the course of understanding and coping with a chronic disease. It can be assumed that spouses and CKD patients have managed to find their ways of coping with implications of the chronic illness, incorporating it in their personal and family life.

Another possible explanation, supported from the researchers of the present study based on clinical experience, could be that spouses, like CKD patients themselves, use defensive mechanisms to deal with their chronic condition. Previous research [53] provided evidence that CKD patients use emotional defensiveness as a coping style which affects their mental and physical aspect of quality of life. It is possible that their spouses, since they live as a psychosocial dyad [27], use a similar coping strategy. It could be assumed that spouses present an ideal image of their self and also of their marriage, either as a psychological defense which allows them to bear with all the chronic implications CKD brings in their life or as an attempt to draw attention away from themselves and maybe more on their spouse.

Spouses reported being the main caregiver and they did not easily ask for help from their entourage (kids, siblings). They reported tendency to delay their health exams and their regular visits to the doctor and postpone advised routine surgeries. These clinical observations match those of Butler et al. [13] who point out that caregiving, except for psychologically burdensome cases, can also be physically burdensome and lead to decreased likelihood to engage in preventive health behaviors. The spouses who felt less burdened from taking care of their partner, despite the amount of time spent taking care of him/her, considered it as a marital debt, or they lived with their spouse as a more closed psychosocial dyad even before the CKD, so the disease did not change their lifestyle.

According to Bialon and Coke [54], there are four main domains which should be studied in order to evaluate and describe the experiences of the caregivers: decline in overall health, role conflict, lack of physical and educational support, and the importance of faith. All the above demonstrate the multilevel influence that caregivers experience, supporting the remark of many researchers that there is need for more educational, physical, and emotional support for individuals who are providing care for sick family members [54]. Caregivers in the early stages of their caregiver experiences are vulnerable to both burden and depression but, although depression and burden may both be forms of caregiver distress, interventions aimed at decreasing burden and depressive symptoms should differ [13, 19].

The results of Khalaila and Cohen [20] reveal a direct relationship between emotional suppression and depressive symptoms, suggesting that caregivers who tend to suppress negative emotions experience poorer mental health. According to the above findings, a key to protection against higher depressive symptoms could be lower emotional suppression (higher expressiveness). Future studies could investigate the implementation of the defensive mechanisms like repression, denial, or idealization in this population.

The spouses do not talk easily about the consequences of their partners' disease in their personal life, which constitutes a challenge for future studies and health professionals. Due to either defensive mechanisms or adjustment to illness, this constitutes a bad prognosis for future deterioration of their health status. In the context of holistic and effective health care, it is important to develop suitable support strategies which will put them in the center of interest, in order to maximize the therapeutic effects of patients' care and also to avoid the deterioration of the spouses' health status. The latter could both worsen the course of illness of the CKD patients and also burden the whole family and the Health Care System (like chronically and financially). Future research could focus on designing intervention strategies on the patents' environment and mostly on their spouses also in the context of Primary Health Care System. Also the relationship between burden and depression in spouses caregivers could be compared between CKD and other chronic diseases and also in larger study groups.

Concerning the limitations of the present study, it should be mentioned that the causality between depression and caregiver burden cannot be defined since, although depression was studied as an outcome, it might have preexisted and could itself have determined outcomes. Also, the limited number of participants should be taken into consideration before generalizing the outcomes of the present study.

## Figures and Tables

**Table 1:** Demographic characteristics of spouse caregivers (S-C) and spouse patients (S-P) with chronic kidney disease (CKD) in two groups.

Demographic characteristics	In all spouses	Spouse (dialysis dependent patient)	Spouse (not dialysis dependent patient)	Difference^2^ (Pearson test)
Gender of spouse, *n* (%)				Pr = 0.979
Male		7 (24.14)	5 (23.81)	
Female		22 (75.86)	16 (76.19)	
Gender of patient, *n* (%)				Pr = 0.979
Male		22 (75.86)	16 (76.19)	
Female		7 (24.14)	5 (23.81)	
Age of spouse				
Mean (SD^1^)	61.84 (11.28)	60.69 (11.13)	63.43 (11.56)	
Min–max	35–84	35–82	43–84	
Age of patient				
Mean (SD)		65.48 (10.54)	66.09 (9.62)	
Min–max		47–83	45–84	
Years related				
Mean (SD)	38.26 (11.70)	38.55 (11.78)	37.85 (11.87)	
Min–max	12–58	12–57	14–58	
Years of CKD				
Mean (SD)	8.76 (7.26)	8.59 (7.79)	9.00 (6.65)	
Min–max		1–40	1–23	
Monthly income				Pr = 0.266
≤500€		3 (10.34)	2 (9.52)	
501–1000€		16 (55.17)	6 (28.57)	
1001–2000€		7 (24.14)	9 (42.86)	
2001–3000€		3 (10.34)	4 (19.05)	
Financial difficulties				Pr = 0.493
None-a few		18 (62.07)	11 (52.38)	
Several-many		11 (37.93)	10 (47.62)	
Education of S-C				Pr = 0.052
Primary education		19 (65.52)	9 (42.86)	
High school		8 (27.59)	5 (23.81)	
Higher education		2 (6.90)	7 (33.33)	
Work status of S-C				Pr = 0.855
Working		7 (24.14)	4 (19.05)	
Not working/housekeeping		9 (31.03)	6 (28.57)	
Pensioners		13 (44.83)	11 (52.38)	

*Note.*  ^1^Standard deviation. ^2^Pearson test chi2 distribution.

**Table 2:** Spouses' mean scores (SD) on burden, depression, anxiety, and quality of life questionnaires in two groups.

Mean (SD^1^)				
Questionnaires	Total sample	Spouses (dialysis dependent patient)	Spouses (not dialysis dependent patient)	Difference of means^7^
ZARIT^2^	27.26 (18.33)	30.62 (1.20)	22.62 (16.39)	0.129
PHQ9^3^	5.64 (4.80)	6.55 (5.50)	4.38 (3.35)	0.115
GAD2^4^	1.90 (1.64)	2.10 (1.74)	1.62 (1.50)	0.309
EQ5D index^5^	0.747 (0.213)	0.769 (0.192)	0.716 (0.241)	0.399
EQ5D VAS^6^	70.86 (16.97)	66.72 (16.60)	76.57 (16,15)	0.042

*Notes.* ^1^Standard deviation. ^2^Zarit Burden Interview. ^3^Patient Health Questionnaire-9. ^4^Generalized anxiety disorder 2. ^5^Health-Related Quality of Life Questionnaire Index. ^6^Health-Related Quality of Life Questionnaire Visual Analogue Scale. ^7^Difference of means was derived using *t*-test.

**Table 3:** Correlation coefficients between spouses' burden, depression, anxiety, and quality of life.

Questionnaires	EQ5D index	EQ5D VAS	PHQ9	GAD2
ZBI^1^	−0.177	−0.482^*∗∗∗*^	0.733^*∗∗∗*^	0.549^*∗∗∗*^
GAD2^2^	−0.490^*∗∗∗*^	−0.405^*∗∗*^	0.763^*∗∗∗*^	
PHQ9^3^	−0.268	−0.417^*∗∗*^		
EQ5D VAS^4^	0.261			

*Notes.*  ^1^Zarit Burden Interview. ^2^Generalized anxiety disorder 2. ^3^Patient Health Questionnaire-9. ^4^Health-Related Quality of Life Questionnaire Visual Analogue Scale. ^*∗∗∗*^*p* ≤ 0.001; ^*∗∗*^*p* ≤ 0.01.

**Table 4:** Multiple linear regression analysis of spouse burden (dependent variable) to depression, stage of chronic kidney disease (dialysis dependent/not dialysis dependent), age, sex, marital satisfaction, education, and employment (*n* = 50).

Independent variable	*B* (St. errors)	*p* value
Depression (PHQ9)	2.577^*∗∗∗*^ (0.385)	**0.000**
Stage (dialysis dependent/not dialysis dependent)	0.077 (3.715)	0.984
Age	0.064 (0.208)	0.759
Sex	−2.044 (4.418)	0.646
VAS-marital satisfaction	2.584 (2.396)	0.287
Secondary education	−2.384 (4.523)	0.601
Higher education	−11.909 (5.924)	0.051
Not working	0.860 (5.127)	0.868
Pensioners	9.364 (5.235)	0.081
Constant	6.724 (14.571)	0.647

*R* ^2^ = 68.2%;^*∗∗∗*^*p* < 0.001.
